# Green-Synthesized vs. Chemical Silver Nanoparticles: A Comparative Study on *S. aureus* Adaptability and Cross-Activity

**DOI:** 10.3390/microorganisms14051114

**Published:** 2026-05-14

**Authors:** Akamu Ewunkem, Josiah Dixon, Jordan Queenie, Uchenna Iloghalu, Franklin Ezeanowai, Sada Boyd

**Affiliations:** 1 Department of Biological Sciences, Winston Salem State University, Winston Salem, NC 27110, USA; jdixon119@rams.wssu.edu (J.D.); jqueenie123@rams.wssu.edu (J.Q.); iloghaluub@wssu.edu (U.I.);; 2 Applied Science, North Carolina A and T State University, Greensboro, NC 27411, USA; cfezeanowai@aggies.ncat.edu

**Keywords:** *S. aureus*, polymorphism, reishi, green synthesis, chemical synthesis, nanoparticles

## Abstract

Rising antibiotic resistance necessitates alternatives such as silver nanoparticles (AgNPs), which exhibit bactericidal activity via multi-target mechanisms (e.g., membrane disruption, ROS production). While resistance to chemically synthesized AgNPs exists, the potential for resistance to green-synthesized AgNPs, such as those from reishi mushroom, is unknown. This study compared *S. aureus* resistance development against both AgNP types using experimental evolution by analyzing genomic and morphological changes. Additionally, this work evaluated potential cross-resistance responses to ionic silver and investigated how adaptation to green-synthesized AgNPs affects sensitivity to chemically synthesized AgNPs (and vice versa). Rapid resistance, along with cross-resistance to silver ions, emerged in bacteria following 14 days of sublethal exposure to silver nanoparticles, regardless of whether they were chemically or biologically synthesized. While green-synthesized AgNPs demonstrated a substantial resistance to chemical variants (*p* < 0.05), the reverse effect was not as strong, and resistant populations showed distinct morphological adaptations. Genomic analysis highlighted convergent hard selective sweeps, identifying common mutations across both chemical and green AgNP-treated populations, with limited unique mutations found for either. These findings enhance our understanding of bacterial resistance mechanisms to nanomaterials, contributing to the development of safer, eco-friendly, and high-efficacy treatments against multidrug-resistant infection.

## 1. Introduction

Rising antibiotic resistance, responsible for significant global mortality, necessitates new treatments such as silver nanoparticles (AgNPs) [1]. AgNPs offer a potent, multi-target alternative to traditional antibiotics, acting through mechanisms such as membrane disruption, DNA damage and ROS production [2,3]. Furthermore, due to electrostatic attraction, AgNPs effectively aggregate on bacteria, releasing ions that cause cell death [4,5]. While capping agents stabilize AgNPs for use against multidrug-resistant pathogens, the synthesis process is paramount, influencing not only the choice of stabilizer but also the economic and ecological impact of the particles.

AgNPs are synthesized via physical (top-down), chemical (bottom-up reduction), and green (biological) methods [6]. While physical methods provide high purity, they are energy-intensive. Chemical reduction (e.g., using citrate/borohydride) is common but often involves toxic agents [7,8]. Green synthesis using extracts or microbes is preferred, offering an eco-friendly, cost-effective, and efficient alternative that yields stable, non-toxic, and highly effective antibacterial agents [9,10].

While AgNPs are hailed as superior, broad-spectrum antimicrobial agents in industrial and medical fields offering alternatives to conventional antibiotics [11,12], their widespread use has created critical ecological and safety issues. The resulting environmental accumulation has highlighted risks of ecotoxicity and, more importantly, the emergence of bacterial resistance to silver itself [13,14]. Despite the efficacy of AgNPs in treating infections, their extensive application necessitates mitigating the development of resistant bacteria [15,16]. A primary mechanism of resistance involves the overexpression of transport proteins (efflux pumps) that remove AgNPs or silver ions from the bacterial cytoplasm [15,16]. To counter rising resistance, this paper outlines mechanisms such as efflux pumps and modified uptake to guide the creation of next-generation agents [17]. Understanding these mechanisms helps overcome bacterial resistance and limits adverse outcomes.

Mushroom-derived synthesis of AgNPs presents a sustainable approach that utilizes natural metabolites as both reductants and capping agents, enhancing both stability and biological functionalities. The structural characteristics are precisely controlled by optimizing parameters (e.g., pH, temperature) to yield optimized antimicrobial, anti-cancer, and wound-healing efficacy.

While early studies in our lab demonstrated *E. coli* 25922 developed rapid resistance to reishi mushroom-mediated green-synthesized AgNPs via genomic changes (Manuscript in preparation), this study investigates *Staphylococcus aureus* adaptation to green-synthesized versus chemically synthesized AgNPs. The goal is to determine comparative resistance rates, uncover the underlying genomic and morphological mechanisms, and assess cross-resistance patterns with chemically synthesized AgNPs and ionic silver. While chemically synthesized AgNP resistance is known [17], this work addresses the limited research regarding bacteria that exhibit resistance to AgNPs produced via green synthesis. Comparative studies between bio-mediated and chemically synthesized AgNPs are vital for optimizing antimicrobial therapies that are both highly effective against resistant strains and environmentally sustainable.

## 2. Materials and Methods

### 2.1. Bacterial Strains and Growth Parameters

The antibacterial efficacy of green and chemically synthesized silver nanoparticles (AgNPs) was evaluated against *Staphylococcus aureus* ATCC# 25923, provided by the Department of Biological Sciences at Winston Salem State University, North Carolina. The bacteria were grown overnight in nutrient broth (Fisher Scientific, NH, Portsmouth, USA) at 37 °C and 160 rpm. The inoculum was standardized to 1.5 × 10^8^ CFU/mL, corresponding to a 0.5 McFarland standard (Fisher Scientific, Hampton, NH, USA).

### 2.2. Nanoparticle Synthesis Routes and Electron Microscopy Techniques (SEM/TEM)

Fresh *Ganoderma lucidum* (also known as reishi mushrooms) were immediately dehydrated (no heat) for three days, then pulverized into particles ~2 mm and stored in double-bagged packaging. For extraction, 100 g of the pulverized sample was mixed with 0.5 L of distilled water in a conical flask, followed by shaking in an incubator at 50 °C and 150 rpm for 48 h. The mixture was centrifuged at 3000 rpm for 10 min and vacuum-filtered (Corning system) at room temperature. The resulting extract was stored in 1 L amber bottles at 4 °C prior to analysis. Adopting the green synthesis approach described by Ewunkem et al. [18], reishi extract was employed to synthesize silver nanoparticles. The procedure involved adding 10 mL of the extract to a 1 mM (AgNO_3_) solution in a 50 mL flask. The mixture was subjected to continuous agitation at room temperature; the transition to a reddish-brown color signaled the reduction of silver ions. Finally, the synthesis was confirmed by measuring the UV-Vis absorbance spectra (200–1000 nm) using a GENESYS 180 spectrophotometer. FTIR measurements were also recorded using a Thermo Fisher Nicolet iS50 ATR-FTIR spectrometer. Spectra were acquired at ambient temperature over a range of 400–4000 cm^−1^ with a resolution of 4 cm^−1^ and an average of 32 scans. Furthermore, the hydrodynamic diameter and zeta potential of the MNP samples were measured at 25 °C using a Malvern Zetasizer Ultra (Malvern, UK). For each 0.7 mL sample, reported values represent the average of three independent measurements. To determine the hydrodynamic diameter, size distribution, and surface charge, the green-synthesized reishi AgNPs were analyzed at 25 °C using a Malvern Zetasizer Ultra (Malvern, UK). All measurements were performed in triplicate for each 0.7 mL sample to ensure reproducibility. The chemically synthesized silver nanoparticles were purchased from Sigma company, St. Louis, MO, USA. The colloidal solution consisted of 20 nm silver nanoparticles chemically synthesized and stabilized with sodium citrate, dispersed in an aqueous buffer at a concentration of 0.02 mg/mL. The size and shape of green and chemical silver nanoparticles were characterized using a JEOL JEM-2100 Plus 200 kV Transmission Electron Microscope (TEM, JEOL USA, Inc.Peabody, MA, USA) capable of (0.19 nm point to point) resolution. Additionally, the morphology of the biosynthesized particles was analyzed via JEOL JSM-IT800 HL SEM (JEOL Ltd., Akishima, Tokyo, Japan) at the Joint School of Nanoscience and Nanoengineering (JSNN), Greensboro, NC, USA. Samples were prepared by drop-coating onto 300-mesh copper grids and observed at 300 kV.

### 2.3. Minimum Inhibitory Concentration (MIC): Green and Chemical Route AgNPs Compared

The minimum inhibitory concentration (MIC) of green-synthesized and chemically synthesized AgNPs was determined by serially diluting them in nutrient broth within 96-well plates. The experimental setup involved diluting bacterial overnight culture to an OD650 nm of 0.05 and plating it. The effects of green-synthesized AgNPs (0–4.5 µM) and chemically synthesized AgNPs (0–47.1 µM) were then assessed across a range of concentrations. All experiments were conducted in triplicate. Turbidity was quantified by measuring the optical density at 650 nm using a GloMax^®^-Multi Microplate reader and clear polyester 96-well plates at both 0 and 24 h, with 0 h values subtracted from 24 h values for statistical evaluation. The sublethal concentrations of silver nanoparticles for selection were established using MIC assay with the ancestral *S. aureus* strain, finding a sublethal concentration 8.0 µM for chemically synthesized AgNPs and 4.5 µM for green-synthesized AgNPs from reishi mushrooms.

### 2.4. Experimental Evolution

To assess adaptation to silver nanoparticles (AgNPs), fifteen *S. aureus* strains, initiated from unique colonies, were cultured in Nutrient Broth (NB) and divided into three groups (n = 5 per group): controls (C1–C5), green-synthesized AgNP treatment (MN1–MN5), and chemically synthesized AgNP treatment (CN1–CN5). Stock cultures were pre-conditioned for seven days via daily 1:100 serial dilutions in 50 mL Erlenmeyer flasks. Experimental cultures were maintained for 24 h at 37 °C in a shaking incubator at 115 rpm, with daily subculturing (0.1 mL transfer into 9.9 mL fresh NB) to simulate continuous selection pressure and ensure consistent population growth from approximately 10^7^ to 10^9^ cells/mL. This replication was designed to eliminate bias, evaluate consistent adaptation to selective pressures, and validate the findings.

### 2.5. Specimen Preparation Procedures for Scanning Electron Microscopy

Morphological changes in nanoparticle-resistant bacteria were analyzed against control and ancestral strains after 14 days of selection. Bacterial suspensions were centrifuged to obtain pellets, which were washed and resuspended in PBS. Cells were fixed using glutaraldehyde, followed by dehydration through a graded ethanol series to preserve structure for visualization via a Carl Zeiss Auriga-BU FIB FESEM.

### 2.6. Structural and Morphological Analysis of Silver Nanoparticles

Nanoparticle characterization included UV-vis spectroscopy (GENESYSTM 180, Fisher Scientific, Portsmouth, NH, USA; 200–1000 nm range) for optical properties and TEM for structural analysis. Chemically synthesized AgNPs were validated using manufacturer-supplied data (Sigma-Aldrich, St. Louis, MO, USA). TEM imaging was conducted at the JSNN (Greensboro, NC, USA) using a JEOL JEM-2100 plus 200 kV microscope (UHR configuration, 0.19 nm resolution) to observe samples drop-coated onto 300-mesh copper grids.

### 2.7. Time-Course Analysis of Phenotypic Growth over 24 h

To evaluate potential pleiotropic effects following 14 days of adaptation to ionic silver, phenotyping assays were performed on evolved populations. The fitness and cross-adaptability of selected populations of green-synthesized AgNPs were tested against increasing concentrations of both chemically and green-synthesized AgNPs, with comparisons drawn against an ancestral *S. aureus* strain grown in Nutrient Broth (NB). Growth was assessed in a 0–45.0 µM range for ionic silver and 0–8.0 µM for green/chemical AgNPs. Bacterial growth was quantified by measuring changes in turbidity (OD650) over 24 h using a GloMax^®^ Multi-Microplate Reader with 98-well polyester plates. The 0 h optical density was subtracted from the 24 h reading to determine the net growth.

### 2.8. Genomic Analysis

Following 14 days of selection, bacterial populations exposed to both green and chemically synthesized AgNPs underwent genomic DNA extraction using the DNeasy 96 PowerSoil Pro QIAcube HT Kit (Qiagen, Redwood City, CA, USA). Libraries were generated using the Illumina DNA Prep tagmentation kit and IDT for Illumina Unique Dual Indexes (Illumina, San Diego, CA, USA). Sequencing was completed on an Illumina NextSeq 2000, with raw data processing (demultiplexing, trimming, and analytics) handled by the integrated DRAGEN v4.2.7 pipeline.

### 2.9. Statistical Analysis

We used SPSS (version 29) to conduct a general linear model analysis on 24 h growth (optical density) across different concentrations of green-synthesized AgNPs, chemically synthesized AgNPs, and ionic silver compared to controls. Bonferroni’s multiple comparisons test determined the significance of mean differences, and GraphPad Prism (version 10) was used to generate plots.

## 3. Results

### 3.1. Characterization of Green-Synthesized and Chemically Synthesized Silver Nanoparticles

**Figure 1:**
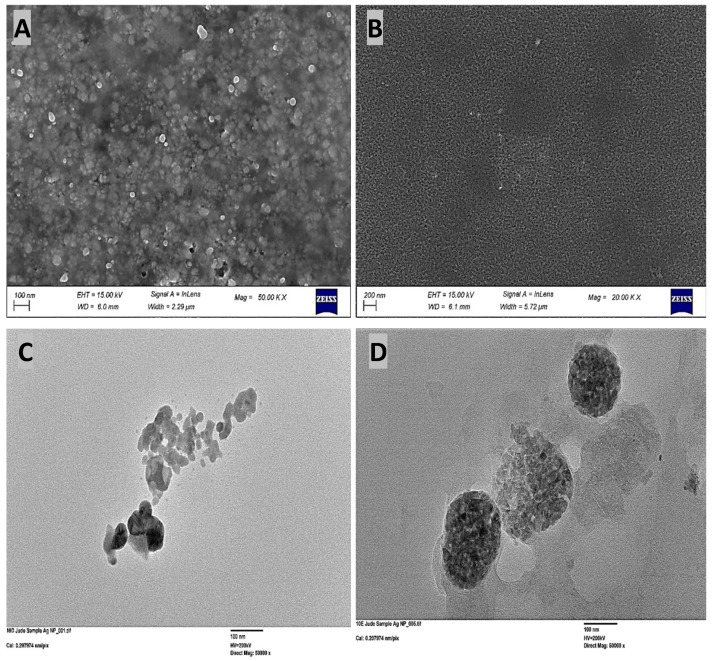
Morphological analysis of silver nanoparticles (AgNPs) via electron microscopy. Scanning Electron Microscopy (SEM) of (**A**) green-synthesized (reishi mushroom) and (**B**) chemically synthesized AgNPs. Transmission Electron Microscopy (TEM) of (**C**) green-synthesized and (**D**) chemically synthesized AgNPs.

Electron microscopy (SEM and TEM) confirmed that both green and chemical synthesis methods successfully produced AgNPs (Figure 1). Imaging revealed that green-synthesized AgNPs were predominantly spherical or near-spherical, with some irregularities, while the chemically synthesized AgNPs were spherical with varying sizes (Figure 1A–D). These morphological findings were supported by spectrophotometric data, which showed both sets of nanoparticles exhibited a characteristic, intense Surface Plasmon Resonance (SPR) peak in the visible spectrum, centered between 440 and 450 nanometers (nm) (See Supplementary Materials Figure S1). Furthermore, zeta potential analysis confirmed a surface charge of −25.76 mV (Supplementary Materials Figure S2) for the reishi mushroom-mediated AgNPs at room temperature, indicating good colloidal stability at a neutral pH. The presence of the functional groups capping AgNPs synthesized using reishi mushroom was analyzed by FTIR and shown in Supplementary Figure S3. Sharp transmittance peaks were observed at 1650.04 and 3400.00 cm^−1^ in the mushroom extract.

### 3.2. Induced S. aureus Resistance to Chemically Synthesized Silver Nanoparticles

**Figure 2:**
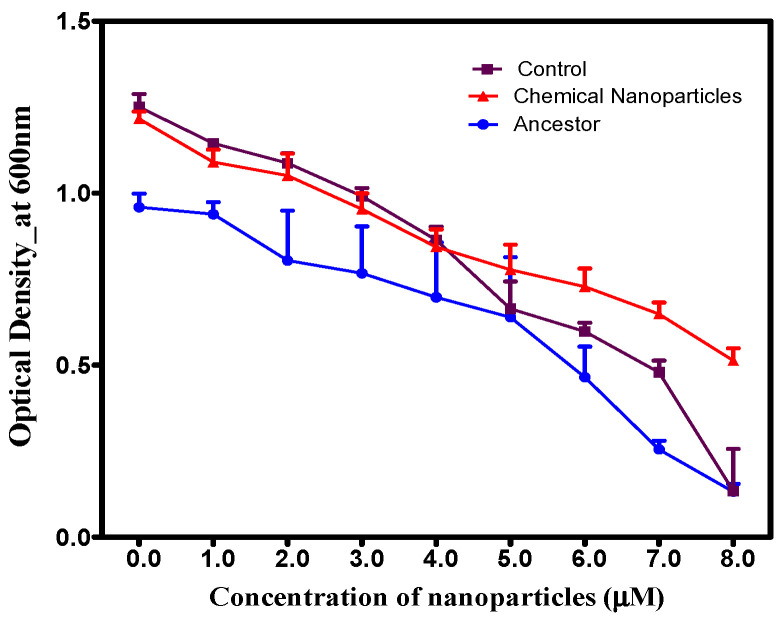
Accelerated growth of adapted *S. aureus* under AgNP stress. Average 24 h population growth comparison after a 14-day evolution experiment. *S. aureus* populations selected under 5.0 µM chemically synthesized AgNP pressure demonstrate enhanced tolerance, with higher growth rates at elevated concentrations relative to control and ancestral strains.

Over a 14-day period, increasing AgNP concentrations led to decreased growth across all populations (Figure 2). Despite this general trend, the specifically selected population demonstrated significantly (*p* < 0.05) higher cell densities than the control/ancestral populations at concentrations ranging from 5.0 to 8.0 µM, indicating enhanced resistance. A significant interaction effect was observed between population and concentration (*F* = 1.328, *p* = 0.194).

### 3.3. Cross-Resistance: Chemically Synthesized Silver Nanoparticles Induce Resistance to Ionic Silver

**Figure 3:**
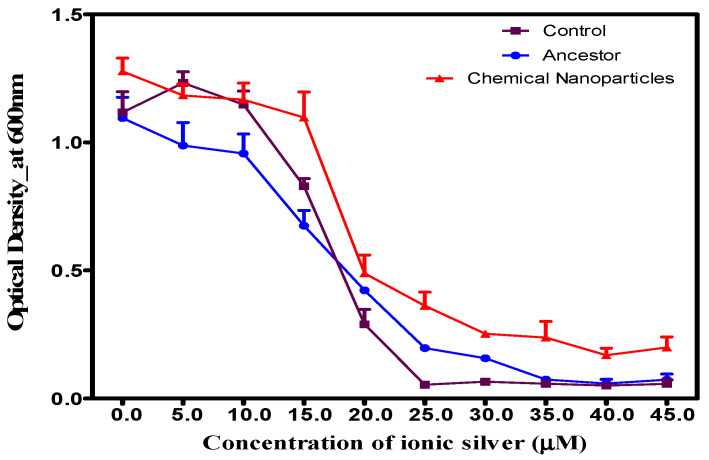
Growth analysis of *S. aureus* adapted to 5.0 µM silver nanoparticles (AgNPs) over 14 days, tested against escalating ionic silver concentrations. Compared to ancestral controls, adapted populations exhibit significantly increased survival and faster growth rates at higher ionic silver levels.

Bacteria that developed resistance to chemically synthesized AgNPs after 14 days of exposure demonstrated cross-resistance to ionic silver. Although growth decreased in all populations as ionic silver concentrations increased, the nanoparticle-adapted bacteria showed significantly (*p* < 0.05) faster and more robust growth after 24 h compared to the unexposed ancestor and control populations (Figure 3). The results indicate a significant interaction between population and concentration.

### 3.4. Cross-Resistance in Chemically Synthesized and Green-Synthesized Silver Nanoparticles

**Figure 4:**
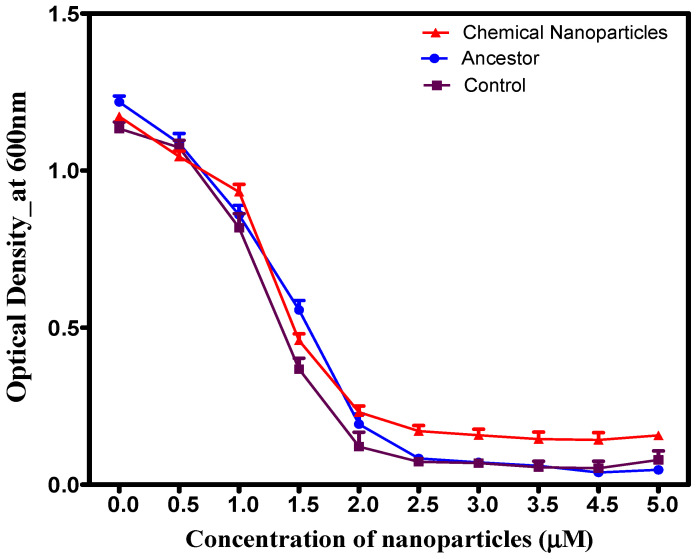
Growth of *S. aureus* populations after 14 days of evolution in increasing concentrations of mushroom-synthesized silver nanoparticles (AgNPs). Populations exposed to chemically synthesized AgNPs exhibited significantly superior growth compared to the control and ancestral populations.

After 14 days, chemically synthesized AgNPs exhibited significant (*p* < 0.05) growth relative to controls and the ancestral strain across all tested reishi mushroom-mediated AgNP concentrations (Figure 4). A significant concentration–population interaction was observed (*F* = 3.571, *p* < 0.001). This growth enhancement was particularly marked at higher concentrations (0.0–5.0 µM), which produced a consistent, steady increase in optical densities.

### 3.5. Induced S. aureus Resistance to Mushroom-Derived Green-Synthesized Nanoparticles

**Figure 5:**
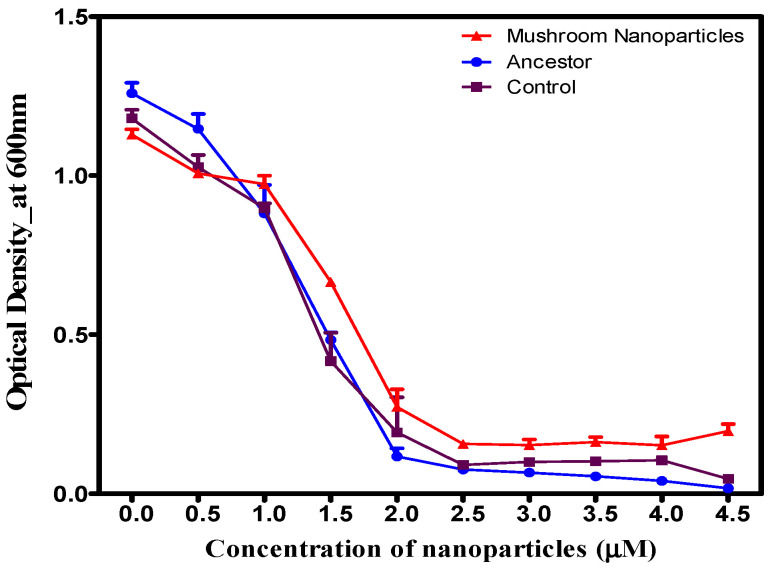
Average 24 h growth of 14-day adapted *S. aureus* populations in reishi-derived silver nanoparticles (AgNPs). *S. aureus* populations exposed to 4.5 µM of reishi-mediated AgNPs for 14 days showed significantly higher growth compared to control (non-exposed) and ancestral (baseline) populations.

Analysis of the 14-day selective pressure experiment (Figure 5) showed that while mushroom-synthesized AgNPs inhibited growth across all populations in a concentration-dependent manner, the specifically selected population displayed significantly superior growth (*p* < 0.0001). The interaction between concentration and population was statistically significant (*F* = 8.964, *p* < 0.001). Specifically, at 1.0–4.5 µM, the adapted population demonstrated significantly higher optical densities compared to control and ancestral groups, highlighting the emergence of resistance.

### 3.6. Cross-Resistance: Reishi Mushroom-Mediated Green-Synthesized Silver Nanoparticles Induce Resistance to Ionic Silver

**Figure 6:**
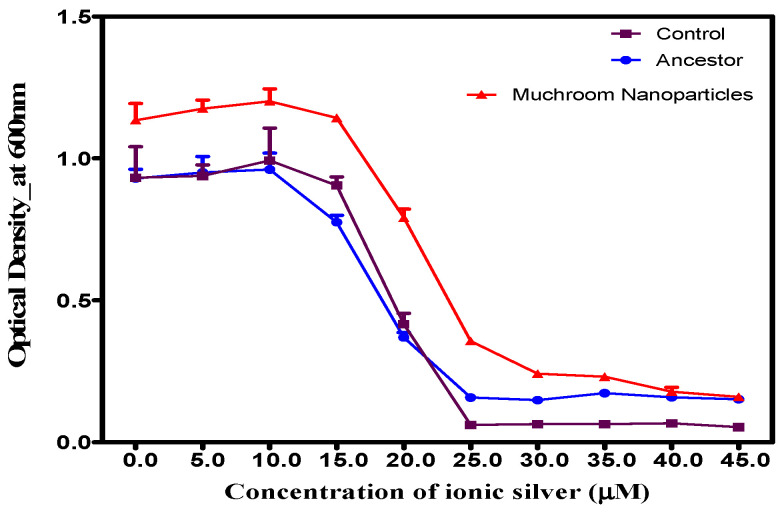
Growth of *S. aureus* populations selected in reishi mushroom-mediated AgNPs, control, and ancestral populations in gradually increasing ionic silver concentrations: the *S. aureus* population evolved in reishi mushroom-mediated AgNPs exhibited significantly (*p* < 0.05) higher growth compared to both the control and ancestral populations.

Resistance mechanisms for AgNPs and ionic silver often overlap or influence each other, so resistance to one can confer resistance to the other, but not always perfectly. When exposed to increasing concentrations of ionic silver (0.0–45.0 µM), populations previously exposed to resihi mushroom-mediated synthesized AgNPs demonstrated significantly (*p* < 0.0001) faster, enhanced, and more robust growth after 24 h compared to both control groups and the original, unexposed ancestral population, as shown in Figure 6. The effect of concentration varied significantly by population (*F* = 3.594, *p* < 0.001).

### 3.7. Cross-Resistance Between Chemically and Green-Synthesized Silver Nanoparticles

**Figure 7:**
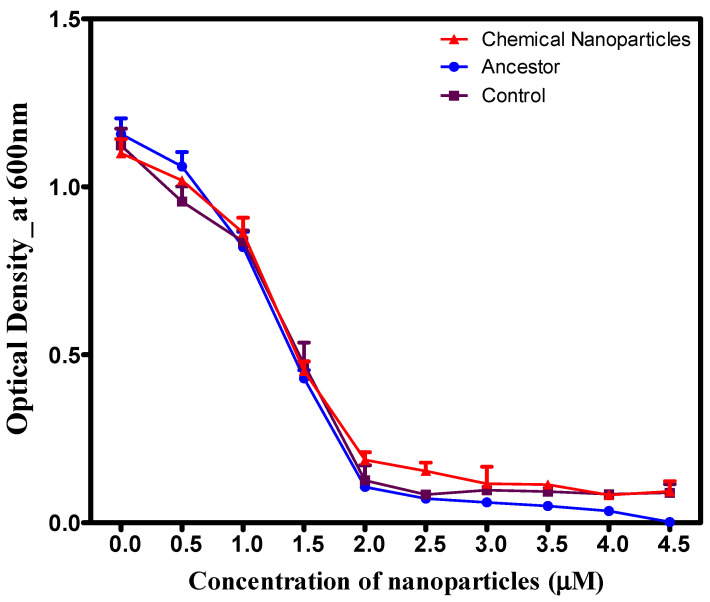
Growth of *S. aureus* populations selected in reishi mushroom-mediated green synthesis, control, and ancestral populations in gradually increasing chemically synthesized AgNPs: The *S. aureus* population evolved in reishi mushroom-mediated nanoparticles exhibited growth comparable to the control and ancestral populations.

Increasing concentrations of reishi-mediated green-synthesized AgNPs resulted in a consistent reduction in growth across all populations (Figure 7). While *S. aureus* previously exposed to chemically synthesized AgNPs demonstrated higher growth rates at elevated concentrations compared to other groups, this difference was not statistically significant (*p* > 0.001) relative to the control and ancestral populations (Figure 7). Concentration levels impacted populations differently, indicating a significant interaction.

### 3.8. Morphological Adaptation and Response of S. aureus to Green and Chemically Synthesized Silver Nanoparticles

**Figure 8:**
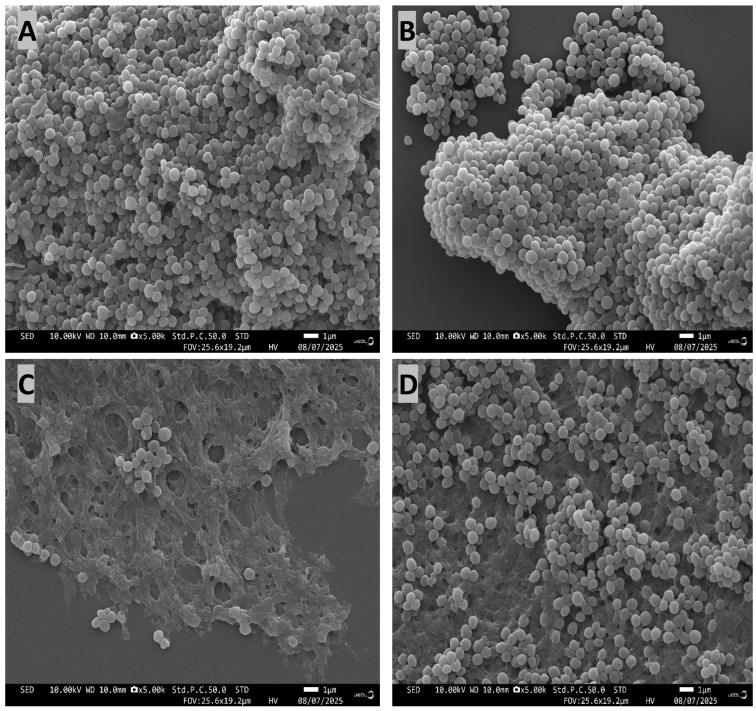
SEM images showing the effect of AgNPs on *S. aureus* morphology after 14 days: (**A**) ancestral cells and (**B**) control cells, both exhibiting smooth surfaces. Cells treated with green-synthesized AgNPs (**C**) and chemically synthesized AgNPs (**D**) show significant disruption, including envelope fragmentation, biofilm damage, and aggregation of particles on the bacterial surface.

*Staphylococcus aureus* typically exhibits a round morphology (Figure 8A,B). Based on Scanning Electron Microscopy (SEM) analysis after 14 days of selection (Figure 8), *S. aureus* cells exposed to silver nanoparticles underwent significant morphological changes from their typical spherical shape to irregular shapes, with the green-synthesized AgNPs (Figure 8C) inducing more severe irregularities compared to the populations selected in the chemically synthesized AgNPs (Figure 8D). While control (Figure 8A) and ancestral (Figure 8B) cells remained consistently spherical and lacked visible biofilm, both silver nanoparticle treatments induced biofilm formation, with a higher degree of associated biofilm observed in cells exposed to the green-synthesized silver nanoparticles.

### 3.9. Genomic Analysis

To identify polymorphisms linked to the selection regime, we performed whole-genome resequencing on all populations after 14 days. By comparing these results to the *Staphylococcus aureus* ATCC 25923 reference genome (NCBI: NZ_CP009361.1), we detected selection-associated genetic variations. Ewunkem et al. [4] detail the ancestral mutations found previously. Exposure to reishi mushroom-mediated AgNPs induced significant adaptation in the bacterial population (MNP1–MNP5), resulting in mutations across 35 distinct genes (Table 1). These genes included *KQ76_RS13020*, *hssR*, *KQ76_RS13825*, *KQ76_RS02145*, *KQ76_RS02150*, *KQ76_RS01495*, *graR*, *KQ76_RS07415*, *KQ76_RS13830*, *KQ76_RS13835*, *KQ76_RS01815*, *KQ76_RS07500*, *KQ76_RS10165*, *mnhD1*, *KQ76_RS12955*, *mbcS*, *KQ76_RS13395*, *icaR*, *pepF*, *pgsA*, *mnmG*, *KQ76_RS00135*, *KQ76_RS12180*, *KQ76_RS10330*, *KQ76_RS13830*, *KQ76_RS13835*, *ylqF*, *KQ76_RS01495*, *KQ76_RS09345*, *pflB*, *sbcD*, *KQ76_RS09345*, *KQ76_RS05490*, *KQ76_RS05495*, *thrS*, *KQ76_RS13700*, *rsp*, *mco*, *KQ76_RS13475* and *KQ76_RS11185*. Notably, a specific mutation emerged with a frequency exceeding 0.4, indicating high prevalence. Furthermore, polymorphisms in genes *KQ76_RS13020*, *hssR*, *KQ76_RS13825*, and KQ76_RS02145/KQ76_RS02150 were universally present across all studied populations. Mutations in *KQ76_RS13825* and *KQ76_RS02145/KQ76_RS02150* were identified in four out of the five replicates. The frequency and genomic distribution of these adaptive mutations are detailed in Table 1, with associated genes listed in Table 2.

Analysis of the bacteria selected under chemical silver nanoparticles (populations CNP1-CNP5) revealed distinct genetic adaptations, including polymorphisms and evidence of selective sweeps, which are detailed in Table 3. Among these, 28 polymorphisms in the CNP-selected populations increased significantly in frequency, from 0.0 to 0.6, within the following genes: *icaR*, *KQ76_RS13020*, *hssR*, *KQ76_RS01495*, *KQ76_RS01815*, *ylqF*, *KQ76_RS10330*, *KQ76_RS13830/KQ76_RS13835*, *sbcD*, *KQ76_RS02145/KQ76_RS02150*, *KQ76_RS12955*, *mnmG*, *rsp*, *KQ76_RS10165*, *KQ76_RS13825*, *folP*, *mco*, *pflB*, *icaR/icaA*, *mnhD1*, *pgsA*, *pepF*, *graR*, *purS*, and *KQ76_RS14600/KQ76_RS09310*. Specific polymorphisms in *icaR*, *icaR/icaA*, *purS*, *and KQ76_RS14600/KQ76_RS09310* were uniquely identified in the chemically synthesized silver nanoparticle-selected bacteria. All population samples showed mutations in *hssR*, *KQ76_RS13020*, and *KQ76_RS01495*, while polymorphisms in mnmG, *KQ76_RS13830/KQ76_RS13835*, and *KQ76_RS10165* occurred in four of five replicates (functional roles listed in Table 2).

Table 4 details 25 putative polymorphisms identified at day 21 across control populations C1–C5. These genetic variations were located within several genes, including *hssR*, *mutS*, *mnmG*, *pflB*, *ylqF*, *pepF*, *folP*, and various others (e.g., *KQ76_RS05075*, *KQ76_RS01495*, *KQ76_RS13020*, *KQ76_RS13825*, *KQ76_RS01815*, *KQ76_RS13830/KQ76_RS13835*, *KQ76_RS02145/KQ76_RS02150*, *KQ76_RS10330*, *KQ76_RS10165*, *KQ76_RS01915*, *KQ76_RS12180*, *KQ76_RS07500*, *KQ76_RS13475*, and *KQ76_RS06970*). Notably, polymorphisms in *KQ76_RS01915* and *KQ76_RS05075* were unique to the control group and were not observed in bacterial populations treated with green or chemically synthesized silver nanoparticles.

## 4. Discussion

The global surge in drug-resistant infections makes the development of novel therapies, specifically silver nanoparticles (AgNPs), critical. AgNPs are effective against bacteria because of their high surface area and release of silver ions [19]. While conventional chemical synthesis exists, eco-friendly “green” synthesis is a growing area of interest [20,21,22]. This research explored how *S. aureus* adapted and developed resistance to both types of AgNPs, looking at both genetic and physical changes to identify unique versus shared resistance pathways.

By utilizing *Ganoderma lucidum* (reishi mushroom) extract for the reduction and stabilization of silver ions, this research explores a sustainable, bio-inspired approach to synthesizing AgNPs. The resulting biogenic nanoparticles were characterized using UV-Vis spectrophotometry, Scanning Electron Microscopy (SEM), and Transmission Electron Microscopy (TEM), and then compared with conventionally produced silver nanoparticles. The successful green synthesis was visually confirmed by the reaction mixture changing from pale yellow brown to reddish-brown within 24 h, a color shift driven by the surface plasmon resonance (SPR) of the particles [22,23]. UV-Vis spectroscopy further verified the formation of the AgNPs by identifying a characteristic SPR peak in the 400–500 nm range [24,25]. Reishi mushroom-mediated AgNPs exhibited a zeta potential of −25.76 mV at room temperature, indicating the formation of negatively charged, highly stable colloids under neutral conditions. This strong negative charge promotes interparticle repulsion, preventing aggregation and ensuring high resistance to agglomeration. FTIR analysis of reishi mushroom-synthesized AgNPs revealed sharp transmittance peaks corresponding to proteins, phenolic compounds, and terpenoids. These biomolecules act as reducing and capping agents, forming a protective layer around the silver nanoparticles to prevent aggregation.

Structural analysis revealed that, unlike conventional chemical methods, which typically produce exclusively spherical nanoparticles, the green synthesis method resulted in particles with diverse, complex morphologies, including rod-like, triangular, hexagonal, and cuboidal shapes. This variation in size and shape arises from the complex mixture of phytochemicals such as triterpenoids, ganoderic acids, and beta [1,2,3] glucans found in the reishi extract, which act as natural reducing and capping agents [18,26]. The potency of nanoparticle antimicrobial activity is driven by minimizing size and optimizing shape [27,28]. Smaller, sub-100 nm particles allow easier penetration of bacterial cells and increased surface reactivity. Meanwhile, asymmetrical geometries, such as triangular plates or nanorods, enhance bactericidal action by increasing physical damage to cellular membranes compared to spherical counterparts [29,30]. The chemically synthesized AgNPs were characterized via UV-Vis spectrophotometry, SEM, and TEM. UV-Vis analysis confirmed AgNP formation through a characteristic surface plasmon resonance (SPR) peak between 400 and 500 nm, while electron microscopy confirmed a spherical morphology. As discussed later, the synthesis method and resultant particle size play a critical role in bacterial adaptation and evolutionary response. These factors influence bacterial adaptation and evolutionary response by attacking multiple, non-specific cellular targets simultaneously.

The structural characteristics of nanoparticles (size and shape), often determined by the method of synthesis (green vs. chemical), play a critical role in determining their efficacy against bacteria and the potential for resistance development. Small, high-surface-area particles generally exhibit superior antimicrobial activity by enhancing contact with bacteria [19]. Furthermore, non-spherical or irregular particles, such as rods or triangles, often display higher bactericidal effects due to their sharper edges, which can damage bacterial membranes and facilitate higher release of silver ions [29,31]. While these enhanced, multi-faceted, or high-surface-area geometries can slow down bacterial adaptation, they are not entirely foolproof, as bacteria can still develop resistance mechanisms, particularly within protective biofilms.

An earlier study in our lab demonstrated that after 24 h of exposure green-synthesized silver nanoparticles exhibited a strong antimicrobial activity against bacteria [32]. These nanoparticles exhibited antimicrobial activity against bacteria by aggregating on the surface of the bacteria, releasing silver into the bacteria, which destroys vital cellular structures [32,33]. Genomic analysis in this study identified mutations in genes related to efflux pump regulation, metabolic pathways, and adaptation, indicating a potential for bacteria to develop resistance to silver nanoparticles [4]. This research examines whether long-term exposure to chemically or green-synthesized AgNPs triggers resistance, while also exploring cross-resistance between both types and against ionic silver to understand bacterial adaptation strategies.

In this study, after 14 days of exposure to green-synthesized AgNPs derived from reishi mushroom, the treated populations exhibited increased AgNP resistance compared to both the control and ancestral populations. In addition, resistance to green-synthesized AgNPs correlated with increased resistance to ionic silver. Conversely, the green-synthesized selected populations showed inferior 24 h growth relative to the controls and ancestor populations in increasing concentrations of chemically synthesized AgNPs. The enhanced growth of the selected population compared to the ancestral control after 14 days in green-synthesized AgNPs can be attributed to adaptive changes, including biofilm formation, genomic alterations, and structural modifications.

Compared to ancestral and control cells, *S. aureus* populations subjected to green-synthesized AgNPs exhibited a marked shift in cell proportion, characterized by significant morphological deviations from a spherical to an irregular shape, coupled with increased biofilm production. This phenomenon indicates that bacterial selection under AgNP stress promotes morphological changes and biofilm formation as protective mechanisms [34,35]. Furthermore, it is established that *S. aureus* mounts a defense against sublethal AgNP concentrations by enhancing the production of biofilm matrix components [36,37,38]. Genetic analysis identified seven specific polymorphisms, exclusively in *S. aureus* populations exposed to reishi-mediated green-synthesized AgNPs, that contributed to resistance. These mutations were localized to key bacterial proteins including the hypothetical protein (*KQ76_RS07415)*, acyl-CoA synthetase (mbcS/KQ76_RS13395), AAA family ATPase (*KQ76_RS00135*), staphylococcal exotoxin (*sem/KQ76_RS09345*), threonine-tRNA ligase (*thrS*), oxidoreductase (*KQ76_RS11185*), and methionine sulfoxide reductase (*KQ76_RS13700*) which are essential for mitigating oxidative stress and adapting to environmental challenges [39,40,41,42,43,44].

Genomic analysis revealed consistent selective sweeps across all four experimental replicates in several key genes, which likely account for the resistance developed by *Staphylococcus aureus* to green-synthesized silver nanoparticles (AgNPs) after 14 days of exposure. These identified genes include: alpha/beta hydrolase fold domain-containing protein (*KQ76_RS13020*), a diverse enzyme family crucial for metabolism, detoxification, and maintaining the cell envelope [45]; DNA-binding heme response regulator (*HssR*), a cytoplasmic regulator that mediates adaptive responses to heme toxicity by activating the *hrtAB* efflux pump, protecting the cell from elevated iron/heme levels [46]; ECF-type riboflavin transporter substrate-binding protein (*KQ76_RS13825*), a membrane component essential for transporting nutrients, aiding in bacterial survival and growth [47]; and hypothetical protein/GNAT family N-acetyltransferase (*KQ76_RS02145/KQ76_RS02150*), proteins involved in regulating bacterial physiology, stress responses, and antibiotic tolerance [48]. In addition, selective sweeps in ECF-type riboflavin transporter substrate-binding protein (*KQ76_RS13825*) and hypothetical protein/GNAT family N-acetyltransferase (*KQ76_RS02145/KQ76_RS02150*) were identified in most of the replicates.

This study also confirmed that resistance to green-synthesized AgNPs directly correlated with enhanced resistance to ionic silver, suggesting cross-resistance driven by shared mechanisms, primarily silver ion release and mutual defense responses. While Gram-negative bacteria (e.g., *E. coli*) typically resist silver through mutations (e.g., *cusS*, *cusR*, *ompR*) and aggregation, Gram-positive bacteria, for example, *S. aureus,* rely on biofilm production as a protective physical barrier [17,37,49]. Our findings demonstrated that *S. aureus* adapted to green-synthesized AgNPs over 14 days by modifying its morphology, strengthening the biofilm matrix, and developing genetic polymorphisms to mitigate metal stress. Ultimately, the enhanced biofilm acts as a shared defense mechanism, restricting the penetration of both ionic silver and green-synthesized AgNPs.

However, the green-synthesized selected populations showed inferior 24 h growth relative to the controls and ancestor populations in increasing concentrations of chemically synthesized AgNPs. Resistance to green-synthesized silver nanoparticles did not confer cross-resistance to chemically synthesized silver nanoparticles primarily because their surface chemistry, capping agents, and mechanism of action differ significantly. Green-synthesized AgNPs from mushrooms are capped by natural bioactive compounds, primarily proteins, enzymes, and polysaccharides, which prevent aggregation by binding to the surface via free amine or carboxyl groups [50,51]. In contrast, chemically synthesized AgNPs are stabilized by synthetic agents such as sodium citrate, which utilize electrostatic repulsion to control size and prevent agglomeration [52]. Due to these distinct differences in surface chemistry and physical properties, bacteria that develop resistance to one type of nanoparticle frequently remain susceptible to the other.

*S. aureus* developed resistance to chemically synthesized AgNPs after only 14 days of exposure, proving that resistance adaptation extends beyond green-synthesized counterparts. This adapted strain showed enhanced growth in high concentration, indicating successful selection. *S. aureus* developed resistance to chemically synthesized AgNPs through morphological adaptations and polymorphism, which allow the bacteria to evade cytotoxic effects by limiting the ability of silver ions to reach cellular targets. When exposed to sublethal concentrations, the bacteria modify their behavior and structure specifically by developing robust biofilms to entrap and aggregate the AgNPs, thus reducing their antibacterial efficacy. Furthermore, this resistance is driven by rapid selective pressure that promotes mutations, causing increased AgNP aggregation, which hinders direct cell contact.

Analysis of bacterial populations exposed to chemically synthesized silver nanoparticles revealed 28 distinct polymorphisms. Among these, three mutations were found universally across all studied populations, indicating a common mechanism of resistance: DNA-binding heme response regulator (*HssR*), a mediator of toxicity responses that activates efflux pumps to protect the cell [46]; alpha/beta hydrolase fold domain-containing protein (*KQ76_RS13020*), which is crucial for maintaining the cell envelope and detoxification [45]; and FTR1 family iron permease (*KQ76_RS01495*), a transport protein that likely contributes to AgNP resistance by altering iron uptake and metabolic defense mechanisms [53]. In four out of five replicates, additional mutations were observed, highlighting common evolutionary paths toward resistance. The S-adenosyl-l-methionine hydroxide adenosyltransferase/YceI family (*KQ76_RS13830/KQ76_RS13835*) is involved in metabolic and adaptive responses, including stress adaptation and antibiotic resistance [54]. tRNA uridine-5-carboxymethylaminomethyl synthesis enzyme (*MnmG*) is associated with modifications in protein synthesis [55] and TrkH family potassium uptake protein (*KQ76_RS10165)*, a membrane component vital for osmotic tolerance and nutrient uptake under stress [56].

Beyond shared mutations, AgNP resistance was further influenced by unique polymorphisms found only in the selected population. Specific, unique mutations were identified in the Ica operon transcriptional regulator (*icaR*) gene, the icaR/icaA intergenic region (−10/−154), Phosphoribosylformylglycinamidine synthase (*purS*), and the *KQ76_RS14600/KQ76_RS09310* locus in bacteria selected via chemically synthesized AgNPs. The icaR gene encodes a TetR-family transcriptional repressor that restricts the production of biofilm-forming polysaccharide intercellular adhesins [57]. Furthermore, the *icaR/icaA* intergenic region (−10/−154) regulates the repression strength of *IcaR* in response to environmental stress, controlling biofilm initiation or inhibition [58]. Finally, *PurS* (phosphoribosylformylglycinamidine synthase) is involved in the fourth step of de novo purine biosynthesis [59]. Purine biosynthesis is crucial for bacterial survival under stress and contributes significantly to resistance by providing energy for cell wall synthesis and enabling metabolic adaptation. Additionally, increased purine production supports biofilm formation and persistence [60].

Populations selected using chemically synthesized AgNPs exhibited superior 24 h growth compared to controls, ancestors, and those treated with green-synthesized AgNPs or ionic silver. This enhanced growth is linked to genetic polymorphisms previously discussed. Furthermore, chemically synthesized AgNPs induce higher resistance because their stable, synthetic capping agents (e.g., borate) provide consistent, high-level ion release, triggering stronger bacterial adaptation than the variable coating of green-synthesized alternatives [61]. These chemically synthesized nanoparticles are also characterized by smaller, more uniform sizes and higher, more consistent reactivity.

Similar to the effects seen with green-synthesized AgNPs, exposure to chemically synthesized AgNPs triggers significant structural alterations in *S. aureus*, resulting in severe peptidoglycan cell wall damage and a loss of their characteristic spherical, smooth (coccus) shape. These morphological changes, which also included reduced cell size and irregular, degraded shapes, allowed *S. aureus* to adapt to the chemically synthesized nanoparticles after a 14-day selection period. Furthermore, comparable modifications in morphology were observed in *E. coli* populations exposed to green-synthesized AgNPs over a 21-day period (manuscript in preparation). These findings align with studies indicating that, over long-term exposure, bacteria alter their shape and structure as an adaptive survival mechanism to mitigate the bactericidal effects of silver nanoparticles [34,35,62].

Analysis revealed shared ancestral-control-selected polymorphisms (*hssR*, *mnmG*, *ylqF*, *KQ76_RS13825*, *KQ76_RS07500*, *KQ76_RS13475*) in both AgNP synthesis types. Furthermore, specific mutations found in both treated and control groups, namely mutS, *pflB*, *pepF*, *folP*, *KQ76_RS05075*, *KQ76_RS01495*, *KQ76_RS01815*, *KQ76_RS13830/35*, *KQ76_RS02145/50*, *KQ76_RS10330*, *KQ76_RS10165*, *KQ76_RS01915*, *KQ76_RS12180*, and *KQ76_RS06970*, were absent in the ancestor. Polymorphisms are shared between ancestral, control, and AgNP-treated populations primarily because both groups are often derived from the same ancestral population and have faced shared parallel evolutionary pressures, both within the experimental environment and during the adaptation process. While AgNP treatment introduces specific selection pressures (e.g., silver toxicity), it does not eliminate all pre-existing genetic variations, and in some cases, the same adaptive mutations are beneficial to both the treated and control groups, albeit perhaps for different reasons. Polymorphisms are typically absent in the ancestral population and present in short-term controls (two-week cultures) because the variants are newly acquired mutations resulting from evolutionary adaptation to a specific environment, rather than standing genetic variation present at the start [2]. Furthermore, the presence of a wide range of polymorphism across all bacterial populations (including the ancestral, control, and those treated populations) suggests that the selection pressure was not intense enough to drive a specific, uniform adaptation or clonal expansion. Using sublethal concentrations of AgNPs for a relatively short duration (two weeks) likely resulted in a “soft sweep” or simply allowed for the maintenance of existing genetic variation.

Several limitations are present in this study. Although green synthesis is safer than chemical methods, the resulting heterogeneous nanoparticles complicate standardized toxicological evaluations. Furthermore, the research overlooks the long-term effects of both types of silver nanoparticles and their potential to cause mutation-driven resistance. A critical lack of gene expression data also limits understanding of how nanoparticle exposure impacts molecular behavior. Finally, the omission of X-ray Diffraction (XRD), a key technique for determining crystallite size, phase composition, and crystal structure, further restricts the characterization of the particles.

## 5. Conclusions

*Staphylococcus aureus* adapts rapidly to silver nanoparticle (AgNP) pressure, with resistance mechanisms dictated by the synthesis method: green-synthesized AgNPs induce quick resistance and cross-resistance to ionic silver, whereas chemically synthesized, uniformly spherical AgNPs trigger distinct, specific resistance. This fast adaptation is driven by quick, structural, and genetic changes, revealing that pre-existing, opportunistic variants are selected rather than entirely new, long-term mutations. This comparative study shows that while both forms of silver nanoparticles are potent antimicrobials, understanding their differing resistance profiles is crucial for designing sustainable, eco-friendly antimicrobial agents that are more resistant to bacterial adaptation. Future studies will involve the long-term experimental evolution of *S. aureus* under sublethal pressure from both reishi-derived green-synthesized silver nanoparticles (AgNPs) and chemically synthesized AgNPs. We aim to characterize the associated genomic changes and analyze gene expression profiles to understand adaptation mechanisms. Additionally, this study will investigate the impact of long-term exposure to these nanoparticles on clinical multidrug-resistant (MDR) *S. aureus* isolates.

## Figures and Tables

**Table 1:** Genomic adaptation of populations selected by mushroom-synthesized silver nanoparticles (14-day exposure).

Gene (16–20)	Position	Mutation	MNP1	MNP2	MNP3	MNP4	MNP5
KQ76_RS13020	2,574,726	G69D (GGC→GAC)	3	4	3	1	1
hssR	2,389,192	R188Q (CGA→CAA)	2	2	2	3	1
KQ76_RS13825	2,746,925	A172V (GCT→GTT)	1	0	1	1	1
KQ76_RS02145/ KQ76_RS02150	460,550	intergenic (+70/−69)	1	1	0	1	0
KQ76_RS01495	339,381	I507L (ATT→CTT)	3	3	3	0	2
graR	673,306	A185T (GCA→ACA)	1	0	0	1	0
KQ76_RS07415	1,542,551	D12V (GAT→GTT)	1	0	0	0	0
KQ76_RS13830/ KQ76_RS13835	2,748,571	intergenic (−255/−127)	1	1	1	1	1
KQ76_RS01815	391,366	S34R (AGT→AGG)	1	1	0	0	2
KQ76_RS07500	1,547,921	S147N (AGT→AAT)	1	0	0	0	0
KQ76_RS10165	1,427,394	I313L (ATT→CTT)	1	0	3	0	0
mnhD1	874,149	L76F (TTA→TTC)	1	0	0	0	1
KQ76_RS12955	2,564,194	A17T (GCA→ACA)	1	0	0	0	0
mbcS/ KQ76_RS13395	2,650,139	intergenic (−101/+93)	1	0	0	0	1
icaR	2,728,076	coding (96/561 nt)	0	1	1	0	0
pepF	940,766	S509R (AGT→AGG)	0	3	0	0	0
pgsA	1,261,441	I19L (ATA→CTA)	0	1	0	0	0
mnmG	2,776,119	L116L (TTG→TTA)	0	1	1	0	0
KQ76_RS00135	34,670	K118K (AAG→AAA)	0	1	0	0	1
KQ76_RS12180	2,408,435	L213L (TTA→CTA)	0	1	0	0	0
KQ76_RS10330	2,061,461	Y3S (TAT→TCT)	0	0	1	0	0
KQ76_RS13830/ KQ76_RS13835	2,748,556	intergenic (−240/−142)	0	0	1	0	1
ylqF	1,213,003	E184E (GAG→GAA)	0	0	1	1	2
KQ76_RS01495	339,390	M510L (ATG→CTG)	0	0	1	4	0
sem/KQ76_RS09345	1,907,997	intergenic (−276/+6)	0	0	0	1	0
pflB	200,288	L193F (TTA→TTC)	0	0	0	1	1
sbcD	1,332,642	N253H (AAC→CAC)	0	0	0	1	0
sem/KQ76_RS09345	1,908,002	intergenic (−281/+1)	0	0	0	1	0
KQ76_RS05490/ KQ76_RS05495	1,123,454	intergenic (+43/−259)	0	0	0	1	1
thrS	1,740,866	E148E (GAG→GAA)	0	0	0	1	0
KQ76_RS13700	2,723,593	L100F (TTA→TTC)	0	0	0	1	0
rsp	2,414,415	C697 (TGC→TGA)	0	0	0	0	1
mco	26,020	N341K (AAC→AAA)	0	0	0	0	1
KQ76_RS13475	2,665,475	S163F (TCT→TTT)	0	0	0	0	1
KQ76_RS11185	2,236,063	R52H (CGT→CAT)	0	0	0	0	1

Notes: Following a 14-day selection process with mushroom-derived silver nanoparticles, 5 bacterial populations (MNP1-MNP5) underwent whole-genome resequencing (breseq v0.30). The tabulated data lists identified genes and mutations, including frequencies.

**Table 2:** Gene catalog and descriptions.

Gene	Gene Product
*KQ76_RS13020*	alpha/beta hydrolase fold domain-containing protein
*hssR*	DNA-binding heme response regulator HssR
*Q76_RS13825*	ECF-type riboflavin transporter substrate-binding protein
*KQ76_RS02145/KQ76_RS02150*	hypothetical protein/GNAT family N-acetyltransferase
*KQ76_RS01495*	FTR1 family iron permease
*graR*	response regulator transcription factor GraR/ApsR
*KQ76_RS07415*	hypothetical protein
*KQ76_RS13830/KQ76_RS13835*	S-adenosyl-l-methionine hydroxide adenosyltransferase family protein/YceI family protein
*KQ76_RS01815*	general stress protein
*KQ76_RS07500*	conserved phage C-terminal domain-containing protein
*KQ76_RS10165*	TrkH family potassium uptake protein
*KQ76_RS06970*	zinc-finger domain-containing protein
*mnhD1*	Na+/H+ antiporter Mnh1 subunit D
*KQ76_RS12955*	D-lactate dehydrogenase
*mbcS/KQ76_RS13395*	acyl-CoA synthetase MbcS/antibiotic biosynthesis monooxygenase family protein
*icaR*	ica operon transcriptional regulator IcaR
*KQ76_RS10330*	accessory gene regulator AgrB
*pepF*	oligoendopeptidase F
*pgsA*	CDP-diacylglycerol--glycerol-3-phosphate 3-phosphatidyltransferase
*mnmG*	tRNA uridine-5-carboxymethylaminomethyl(34) synthesis enzyme MnmG
*KQ76_RS00135*	AAA family ATPase
*KQ76_RS12180*	magnesium transporter CorA family protein
*KQ76_RS01495*	FTR1 family iron permease
*sem/KQ76_RS09345*	staphylococcal enterotoxin type M/exotoxin beta-grasp domain-containing protein
*ylqF*	ribosome biogenesis GTPase YlqF
*pflB*	formate C-acetyltransferase
*sbcD*	exonuclease subunit SbcD
*KQ76_RS05490/KQ76_RS05495*	hypothetical protein/IS1182-like element ISSau3 family transposase
*thrS*	threonine--tRNA ligase
*KQ76_RS13700*	peptide-methionine (S)-S-oxide reductase
*rsp*	AraC family transcriptional regulator Rsp
*mco*	multi-copper oxidase Mco
*KQ76_RS01495*	FTR1 family iron permease
*KQ76_RS13475*	glutathione peroxidase
*mbcS/KQ76_RS13395*	acyl-CoA synthetase MbcS/antibiotic biosynthesis monooxygenase family protein
*KQ76_RS11185*	NADP-dependent oxidoreductase
*KQ76_RS10330*	accessory gene regulator AgrB
*KQ76_RS13830/KQ76_RS13835*	S-adenosyl-l-methionine hydroxide adenosyltransferase family protein/YceI family protein
*KQ76_RS02145/KQ76_RS02150*	hypothetical protein/GNAT family N-acetyltransferase
*KQ76_RS12955*	D-lactate dehydrogenase
*folP*	dihydropteroate synthase
*icaR/icaA*	ica operon transcriptional regulator IcaR/poly-beta-1,6 N-acetyl-D-glucosamine synthase IcaA
*KQ76_RS14600/KQ76_RS09310*	helix-turn-helix domain-containing protein/DUF6978 family protein
*purS*	phosphoribosylformylglycinamidine synthase subunit PurS
*KQ76_RS05075*	cytochrome d ubiquinol oxidase subunit II
*mutS*	DNA mismatch repair protein MutS
*KQ76_RS01915*	superantigen-like protein SSL10
*KQ76_RS07500*	conserved phage C-terminal domain-containing protein
*fdhD*	formate dehydrogenase accessory sulfurtransferase FdhD

**Table 3:** Genomic adaptation of populations selected by chemically synthesized silver nanoparticles (14-day exposure).

Gene (21–25) Chemical	Position	Mutation	CN1	CNP2	CN3	CN4	CN5
icaR	2,728,076	coding (96/561 nt)	1	1	0	0	1
*KQ76_RS13020*	2,574,726	G69D (GGC→GAC)	3	3	2	2	3
*hssR*	2,389,192	R188Q (CGA→CAA)	3	3	3	1	3
*KQ76_RS01495*	339,381	I507L (ATT→CTT)	2	3	3	3	2
*KQ76_RS01815*	255.1/38.8	L40R (CTA→CGA)	2	0	2	2	0
*ylqF*	1,213,003	E184E (GAG→GAA)	1	0	0	2	1
*KQ76_RS10330*	2,061,461	Y3S (TAT→TCT)	1	0	1	1	0
*KQ76_RS13830* */KQ76_RS13835*	2,748,571	intergenic (−255/−127)	1	0	1	0	1
*sbcD*	1,332,654	I257L (ATT→CTT)	1	1	0	2	0
*KQ76_RS13830/* *KQ76_RS13835*	2,748,573	intergenic (−257/−125)	1	0	0	0	0
*KQ76_RS02145* */KQ76_RS02150*	460,550	intergenic (+70/−69)	1	1	0	1	1
*KQ76_RS12955*	2,564,194	A17T (GCA→ACA)	1	0	0	0	0
*KQ76_RS13830* */KQ76_RS13835*	2,748,556	intergenic (−240/−142)	1	1	1	0	1
*mnmG*	2,776,119	L116L (TTG→TTA)	0	1	1	1	1
*rsp*	2,413,415	coding (1091/2106 nt)	0	1	0	0	0
*KQ76_RS10165*	2,037,141	V315L (GTA→TTA)	0	1	2	1	1
*KQ76_RS13825*	2,746,925	A172V (GCT→GTT)	0	1	0	0	1
*folP*	504,457	V222I (GTA→ATA)	0	1	0	0	0
*mco*	26,020	N341K (AAC→AAA)	0	1	0	0	0
*pflB*	200,288	L193F (TTA→TTC)	0	1	0	0	0
*icaR/icaA*	2,728,181	intergenic (−10/−154)	0	0	1	0	0
*mnhD1*	874,149	L76F (TTA→TTC)	0	0	1	0	0
*pgsA*	1,261,441	I19L (ATA→CTA)	0	0	1	0	0
*pepF*	940,717	L493 (TTA→TGA)	0	0	0	2	0
*graR*	673,306	A185T (GCA→ACA)	0	0	0	0	1
*KQ76_RS14600* */KQ76_RS09310*	1,901,738	intergenic (+1/−100)	0	0	0	0	1
*purS*	1,027,271	A87T (GCA→ACA)	0	0	0	0	1
*KQ76_RS14600* */KQ76_RS09310*	1,901,745	intergenic (+8/−93)	0	0	0	0	1

Notes: Following a 14-day selection process with chemically synthesized silver nanoparticles, 5 bacterial populations (CN1–CN5) underwent whole-genome resequencing (breseq v0.30). The tabulated data lists identified genes and mutations, including frequencies.

**Table 4:** Day 21 single-nucleotide polymorphism analysis in control populations.

Gene (26–30)	Position	Mutation	C1	C2	C3	C4	C5
KQ76_RS05075	1,048,213	pseudogene (686/1021 nt)	1	1	1	0	0
hssR	2,389,192	R188Q (CGA→CAA)	1	3	3	2	2
KQ76_RS01495	339,381	I507L (ATT→CTT)	2	3	3	2	1
KQ76_RS13020	2,574,726	G69D (GGC→GAC)	2	2	3	3	1
KQ76_RS13825	2,746,925	A172V (GCT→GTT)	1	1	1	1	1
KQ76_RS01815	391,366	S34R (AGT→AGG)	2	2	2	2	2
KQ76_RS13830 /KQ76_RS13835	2,748,556	intergenic (−240/−142)	1	0	1	1	1
KQ76_RS13830 /KQ76_RS13835	2,748,571	intergenic (−255/−127)	1	0	1	1	1
mutS	1,275,400	K479K (AAG→AAA)	1	0	0	0	0
KQ76_RS02145 /KQ76_RS02150	460,550	intergenic (+70/−69)	1	0	0	0	0
KQ76_RS10330	2,061,461	Y3S (TAT→TCT)	1	1	1	1	0
mnmG	2,776,119	L116L (TTG→TTA)	1	0	1	0	0
KQ76_RS10165	2,037,141	V315L (GTA→TTA)	1	3	1	0	2
KQ76_RS01915	411,304	K77K (AAG→AAA)	1	0	0	0	1
pflB	200,288	L193F (TTA→TTC)	1	0	0	1	3
ylqF	1,213,003	E184E (GAG→GAA)	0	1	0	3	0
pepF	940,717	L493 (TTA→TGA)	0	1	0	0	0
KQ76_RS12180	2,408,435	L213L (TTA→CTA)	0	1	0	0	3
KQ76_RS07500	1,547,921	S147N (AGT→AAT)	0	1	0	1	0
KQ76_RS13475	2,665,475	S163F (TCT→TTT)	0	0	1	0	0
folP	504,457	V222I (GTA→ATA)	0	0	1	1	0
KQ76_RS06970	1,427,399	L5F (TTA→TTC)	0	0	0	2	0

Notes: Following a 14-day selection process, 5 control populations (C1–C5) underwent whole-genome resequencing (breseq v0.30). The tabulated data lists identified genes and mutations, including frequencies.

## Data Availability

Data are contained within the article.

## Supplementary Materials

The following supporting information can be downloaded at: https://www.mdpi.com/article/10.3390/microorganisms14051114/s1. Figure S1: UV-Vis absorption spectra confirming the green synthesis of silver nanoparticles (AgNPs) mediated by *Ganoderma lucidum* (Reishi) extract, showing the characteristic Surface Plasmon Resonance (SPR) peak; Figure S2: Zeta potential distribution of green synthesized silver nanoparticles (AgNPs) mediated by *Ganoderma lucidum* (Reishi) extract, indicating a surface charge of −25.76 mV and suggesting high colloidal stability. MNP indicates silver nanoparticles synthesized via mushroom extract; Figure S3: FTIR spectra of *Ganoderma lucidum* (Reishi) extract-mediated AgNPs, highlighting functional groups responsible for the reduction, capping, and stabilization of the silver nanoparticles.
