# Supplementary material for: Green-Synthesized vs. Chemical Silver Nanoparticles: A Comparative Study on S. aureus Adaptability and Cross-Activity
Source: Microorganisms. 2026 May 14;14(5):1114. doi: 10.3390/microorganisms14051114 (PMC13210130; doi:10.3390/microorganisms14051114)
Supplement: Supplementary file 1 [file microorganisms-14-01114-s001.zip › microorganisms-4257960-supplementary.pdf]

Supplementary Materials

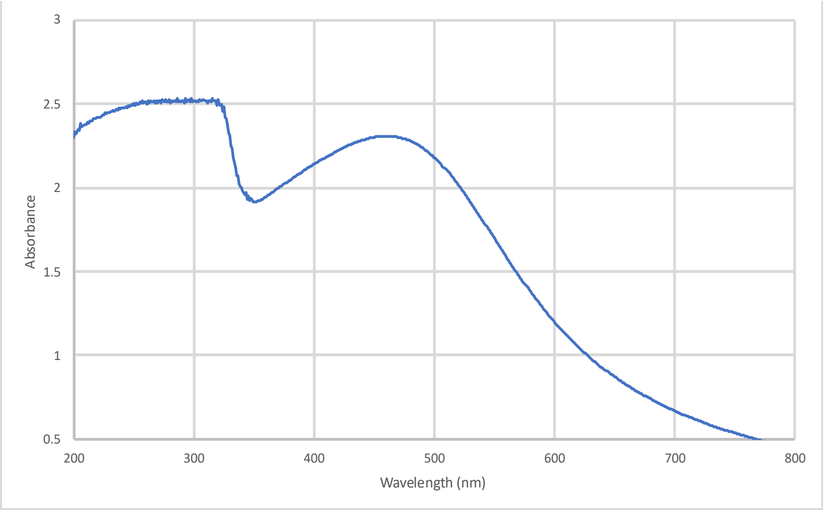

Figure S1. UV-Vis absorption spectra confirming the green synthesis of silver nanoparticles (AgNPs) mediated by *Ganoderma lucidum* (Reishi) extract, showing the characteristic Surface Plasmon Resonance (SPR) peak.

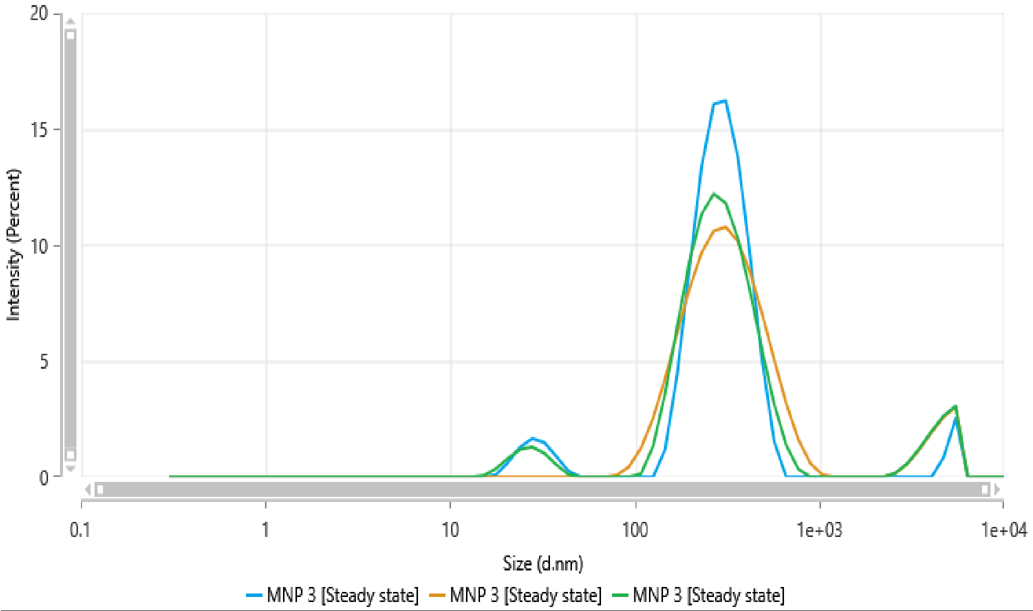

Name	Mean	Standard Deviation	RSD	Minimum	Maximum
Zeta Potential (mV)	-25.76	0.6472	2.512	-26.32	-25.05
Conductivity (mS/cm)	0.326	0	0	0.326	0.326
Wall Zeta Potential (mV)	-22.8	3.016	13.23	-26.23	-20.58
Quality Factor	3.483	0.4153	11.92	3.032	3.848
Zeta Peak 1 Mean (mV)	-25.76	0.6472	2.512	-26.32	-25.05

Figure S2. Zeta potential distribution of green synthesized silver nanoparticles (AgNPs) mediated by *Ganoderma lucidum* (Reishi) extract, indicating a surface charge of −25.76 mV and suggesting high colloidal stability. MNP indicates silver nanoparticles synthesized via mushroom extract

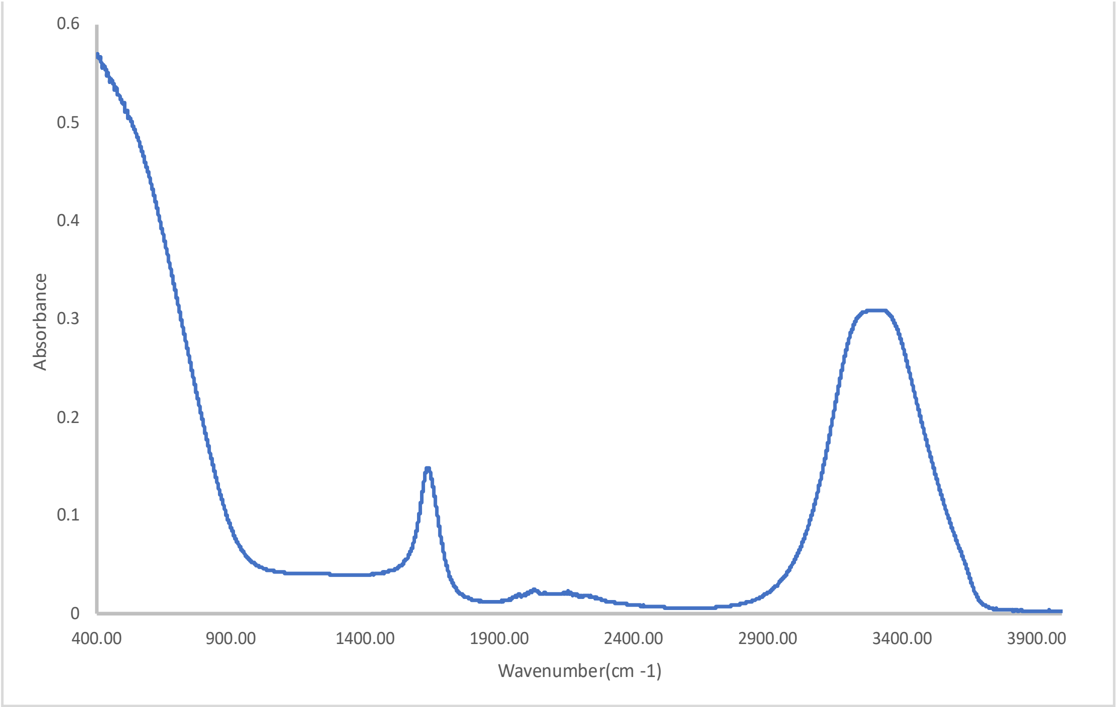

Figure S3. FTIR spectra of Ganoderma lucidum (Reishi) extract-mediated AgNPs, highlighting functional groups responsible for the reduction, capping, and stabilization of the silver nanoparticles.
